# Characteristics of Components and Density of Rigid Nanoclay-Filled Medium-Density Polyurethane Foams Produced in a Sealed Mould

**DOI:** 10.3390/polym15153228

**Published:** 2023-07-28

**Authors:** Ilze Beverte, Ugis Cabulis, Janis Andersons, Mikelis Kirpluks, Vilis Skruls, Peteris Cabulis

**Affiliations:** 1 Institute for Mechanics of Materials, University of Latvia, 3 Jelgavas St., LV-1004 Riga, Latvia; janis.andersons@pmi.lv (J.A.); skruls@pmi.lv (V.S.); 2 Latvian State Institute of Wood Chemistry, 27 Dzerbenes St., LV-1006 Riga, Latvia; ugis.cabulis@kki.lv (U.C.); mikelis.kirpluks@kki.lv (M.K.); peteris@ritols.lv (P.C.)

**Keywords:** polyurethane foams, medium density, sealed mould, filler, nanoclay, composite, mass, volume, concentration

## Abstract

The characteristics of rigid, nanoclay-filled, medium-density NEOpolyol-380 polyurethane foams components can be estimated when two conditions are met: (1) the foam blocks are produced in a sealed mould; and (2) the mass of the reacting mixture is kept constant. It was shown that, with an increase in filler concentration, the total mass of the filled polymeric network stays constant, but the total volume reduces; the higher the ratio of density of the exfoliated nanoclay platelets and polymer, the higher the volume reduction of the polymeric network. Experimental data of polyurethane foam block mass were acquired at concentrations η = 0%, 0.25%, 0.5%, 1%, 2%, 3% and 5% from the mass of a filled reacting mixture. Foam-density dependence in the uniform zone and in the side-sections of the produced blocks on the: (1) mass of the blocks; and (2) the concentration of the filler was analysed. The study demonstrated that the correlation of the specimens’ density with the foam block mass is much higher than that of the filler concentration.

## 1. Introduction

Rigid medium-density polyurethane (PU) foams, with a density of 200–250 kg/m^3^, are used in test milling, in the construction of simple negative moulds and laminating moulds, in design studies and modelling, and as substructures for model pastes etc. [1,2]. The foams are used in diverse applications, as impact-mitigating components in the automotive industry and as structural materials in various engineering solutions [3,4,5]. The foams are used as encapsulants of electronic components to mitigate harsh thermal and mechanical environments as well as to provide electrical isolation [6,7].

Nanoclays like montmorillonite are popular nanofillers for many polymeric systems because they can improve thermal stability and flame retardancy, are lightweight and have high compressive strength [8,9]. Well-dispersed nanoparticles act as nucleation sites and facilitate the formation of bubbles, thus leading to a reduction in foam cell size [10,11,12,13]. The exfoliated clay nanoplatelets enhance the foam’s mechanical properties and reduce gas diffusivity in the walls of cells (the barrier effect) [12,13,14,15,16,17].

Nanoclays, such as hydrophilic bentonite, MMT K10, Cloisite-10A, Cloisite15A, Cloisite-30B, etc., are filled either into polyol systems or isocyanate or a mixture of both at a certain concentration (mostly ≤10 wt% of polyol or isocyanate mass) and then processed by lab mixers, ultrasonic cavitation, high shear mixers, etc. to disperse and exfoliate the clay platelets [8,9,10,11,12,13,14,15,16,17,18]. The percentage of clay in the mass of polyol or isocyanate can be recalculated relative to the mass of the liquid reacting mixture [3]. A rapid increase in viscosity of the liquid reacting mixture is marked with an increase in the clay content [10,11,12,13,14,15]. The greatest improvements in the foam’s physical and mechanical properties were reported at nanoclay filler concentrations <5% [3,8].

When the clay platelets are dispersed and exfoliated in the polymer matrix, the platelets themselves provide the modifications. Excluding the interlayer water (6 wt.%) and volume of the interlayer spacing (20 vol.%) and adjusting the density 2600 kg/m^3^ of bulk clay, the density of exfoliated clay platelets is deduced as 3067 kg/m^3^ [19,20]. The platelet density can be estimated from the experimentally measured gallery area, which is 310 m^2^g^−1^. When the effective platelet thickness is 0.98 nm, the density equals 3292 kg/m^3^, which is comparable to that calculated from the density of bulk clay. It can be seen that the density of the exfoliated nanoclay platelets is nearly 2.6 times greater than the density of monolithic polyurethane, at 1280 kg/m^3^.

In an open mould, in a free rise, mainly anisotropic PU foams can be produced [1,21,22,23]. The degree of anisotropy of nanoclay-filled PU foams differs from that of neat foams, because the foams rise to different heights [13,24]. The physical/mechanical properties of neat and filled free-rise PU foams differ not only due to their fillings, but also due to different anisotropy degrees. This hinders an accurate evaluation of the impact of the filling on PU foam properties.

In a sealed mould, at a high overpressure, nearly isotropic PU foams can be produced [3,25,26]. In [6], the mixed liquid composition of PU foams was poured into cylindrical moulds to produce foams with densities of 100 kg/m^3^… 400 kg/m^3^. Then, the mould was sealed, and the foam was allowed to expand to fill the sealed mould to a density that exceeded the expected free-rise density by approximately 1.75 times. In [12] the liquid reacting mixture of PU foams, with an expected density of 240 kg/m^3^, was poured into a preheated mould, constructed by two aluminium plates on both sides of an aluminium frame. In [3,25,26] the reacting mixture of NEOpolyol-380 PU foams, both neat and nanoclay-filled, was poured into a stainless-steel mould and the mould was sealed. The overpressure in the sealed mould was p_ov_ ≈ 1.7 atm, which allowed for the production of nearly isotropic PU foam blocks. At the same time, it remains unclear how the mass and volume of the filled PU foam components (polymer, gaseous phase and filler) change when nanoclay is filled into the reacting mixture at a condition of m_0_ = const. and the mixture is formed in a sealed mould (m_0_—mass of the filled reacting mixture).

In the production of nanoclay-filled PU foam blocks, filler concentrations are varied on purpose, but the mass of the reacting mixture, poured into the mould, varies unintentionally from block to block for different technological reasons [1,25,27]. Correct estimation of the average density in different locations of a PU foam block is dependent on: (1) the actual mass of the block; and (2) the filler concentration. The estimation is important for concentrations of filler <5%, where considerable improvements of physical/mechanical properties have been identified [3,8].

The main aim of this study was to estimate the characteristics—mass and volume—of rigid nanoclay-filled medium-density NEOpolyol-380 PU foam components (polymer, gaseous phase, and filler), when the foam was made in a sealed mould. The study showed that when the foam blocks are in a condition of constant mass, the summary mass of the polymeric network, filled width nanoclay platelets, stays constant, but the summary volume decreases. The results also showed that he experimentally determined PU foam density in the uniform zone and in the side section of blocks is dependent on the: (a) mass of PU foam blocks; and that (b) the filler concentration must be analysed as well. An approximation of experimental data trends revealed that the correlation between the density of the specimens and the blocks’ mass was high, but that the correlation with the filler concentration was insignificant.

## 2. Materials and Methods

### 2.1. Raw Materials and Production of PU Foams

Rigid closed-cell medium-density NEOpolyol-380 polyurethane foam blocks shaped as truncated pyramids were produced in a sealed steel mould according to the formulation in Table 1. The pMDI and polyol components were weighed and mixed with a mechanical stirrer at 2000 rpm for 15 s. The mould was preheated to 50 °C and an appropriate amount of the reacting mixture was poured into it; then, the mould was sealed. The raw materials and production technologies are given in detail in [25,26].

The recycled APP NEOpolyol-380 can be considered a sustainable raw material because it is produced from industrial PET waste. The formulation comprises ~15% of recycled materials. Cloisite-30B was added as a nanoclay filler in concentrations of η = 0.25%, 0.5%, 1%, 2%, 3% and 5% from the mass of the filled reacting mixture, which was calculated so as to produce PU foam blocks of an apparent overall density ≈250 kg/m^3^ (ISO 845:2006) [28].

When nanoclay is filled into a monolithic polymer or plastic foam, the properties of the composite are determined by the degree of dispersion, intercalation, and exfoliation of the filler. Intercalation and exfoliation of the nanoclay Cloisite-30B monolayers was evaluated via the basal spacing by X-ray diffraction (XRD), at a 5 wt.% concentration of nanoclay (from the mass of NEOpolyol-380) in “Cloisite-30B/NEOpolyol-380” dispersions. The methodology of the XRD analysis is described in [25,26], which deal with the same nanoclay-filled NEOpolyol-380 PU foams.

The technological target was to keep the mass of the filled reacting mixture constant for all concentrations at m_0_ = 250 g = const [25,26]. The mass m_0_ was calculated to produce PU foams of a density exceeding the corresponding free-rise density by approximately 1.75 times. An overpressure occurs in the sealed mould which facilitates the production of nearly isotropic PU foams.

### 2.2. PU Foam Blocks and Density Specimens

Mass of the 7 produced filled NEOpolyol-380 PU foam blocks (η = 0%, 0.25%, 0.5%, 1%, 2%, 3% and 5%) was measured. The relative difference between the target mass m_0_ = 250 g = const. and that of the n-th filled block m_n_ was calculated:

(1) $$R_{n}=\frac{\left|{∆m}_{n}\right|}{m_{0}};\;\mathrm{where}\;∆m_{n}=m_{0}-m_{n}\;\mathrm{and}n=1,\;2,\;\ldots ,\;7.$$

**Figure 1:**
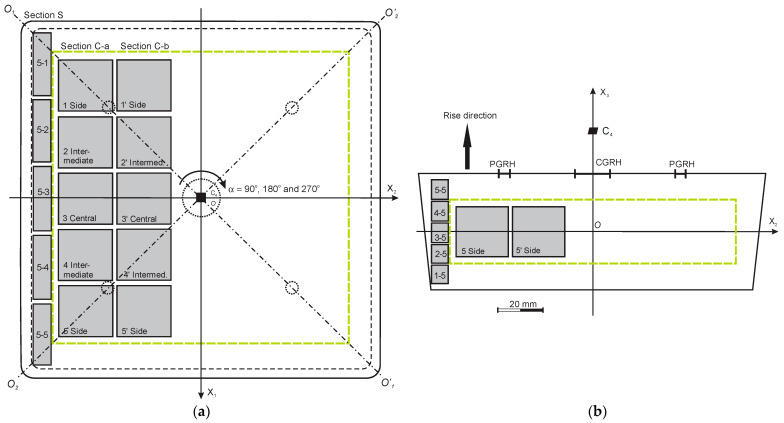
A PU foam block: (**a**) top view; and (**b**) side view; PGRH—peripheral gas-release holes and CGRH—central gas-release hole. The green rectangles enclose a zone of comparatively uniform density; α—angle of rotation around axis OX_3_; O_1_O’_1_ and O_2_O’_2_—diagonal axes of symmetry.

A zone of comparatively uniform density was outlined in the blocks, based on: (1) Visual estimation of the cutting surfaces of the blocks and (2) The 4-th order rotational symmetry C_4_ around axis OX_3_ of the blocks, Figure 1. Two Sections of density specimens were made in the uniform zone: “C-a” and “C-b”. Five cubic specimens, 22 × 22 × 22 mm, were made from each Section and their apparent density was determined (ISO 845:2006, further—density).

Locations of the cubic specimens in foam blocks were denoted as “Side” (1, 1′ and 5, 5′), “Intermediate” (2, 2′ and 4, 4′) and “Central” (3, 3′), Figure 1. The specimens located symmetrically to the plane X_2_OX_3_ were considered to be in similar foaming conditions: 1 and 5, 2 and 4 as well as 1′ and 5′, 2′ and 4′. Average density of the specimens from similar locations was calculated:

(2) $$\rho_{1,5}=\frac{1}{2}\left(\rho_{1}+\rho_{5}\right),\rho_{2,4}=\frac{1}{2}\left(\rho_{2}+\rho_{4}\right)\;\mathrm{and}\;\rho_{1\prime ,5\prime }=\frac{1}{2}\left(\rho_{1\prime }+\rho_{5\prime }\right),\rho_{2\prime ,4\prime }=\frac{1}{2}\left(\rho_{2\prime }+\rho_{4\prime }\right).$$

To investigate the density distribution at the side of a block, Section S of the parallelepiped-shaped specimens was constructed next to Section C-a of the cubic specimens (Figure 1). Section S was cut into 25 specimens with the dimensions of 9 mm × 8 mm × 25 mm. Density was measured for the specimens from columns 1, 2 and 3. Taking into account the symmetry of the block with respect to the plane X_2_OX_3_, it was assumed that the density in columns 4 and 5 was equal to that in columns 2 and 1 (Supplementary Materials, Table S1). For each block, the average densities of the 9 central and 16 perimetral side specimens, as well as the corresponding absolute and the relative density differences, were calculated:

(3) $$\rho_{9}=\frac{1}{9}\sum_{i=1}^{9}\rho_{i},\rho_{16}=\frac{1}{16}\sum_{i=1}^{16}\rho_{i};$$

(4) $$∆\rho_{16,9}={(\rho }_{16}-\rho_{9})\;\mathrm{and}\;R_{16,9}=\frac{∆\rho_{16,9}}{\rho_{9}}$$

To estimate the difference in the average densities of the (a) 9 central side specimens and (b) 16 perimetral side specimens and in the adjacent uniform part of the block (the three cubic specimens No 2, 3 and 4 from Section C-a), the absolute and the relative density differences were calculated:

(5) $$∆\rho_{9,2-4}=\left(\rho_{9}-\rho_{2-4}\right);\;R_{9,2-4}=\frac{∆\rho_{9,2-4}}{\rho_{2-4}}\;\mathrm{and}$$

(6) $$∆\rho_{16,2–4}=\left(\rho_{16}-\rho_{2–4}\right);\;R_{16,2–4}=\frac{∆\rho_{16,2–4}}{\rho_{2–4}};\;\mathrm{where}\;\rho_{2–4}=\frac{1}{3}\sum_{i=2}^{4}\rho_{i}.$$

ρ_2–4_ is the average density of the cubic specimens No 2, 3 and 4 from the Section C-a, located next to the nine central side specimens, Figure 1.

The dependence of densities ρ_1,5,_ ρ_1′,5′_, ρ_2,4_, ρ_2′,4′_, ρ_3_, ρ_3′_ and ρ_9_, ρ_16_, ρ_2–4_ on the: (1) actual mass of the blocks; and (2) the concentration of the filler was analysed. Numerical calculations were made for several sets of input data. The approximating equations for the relationships “m—ρ” and “η—ρ” were derived and the coefficients of correlation were compared. The increase rate Δρ/Δm of the average density with respect to the blocks’ mass was calculated in different locations of blocks. The experimental data were compared with the theoretical estimations of density in the blocks.

## 3. Theoretical

### 3.1. The Main Assumptions

Nanoclay-filled PU foam is a “Polymer—gas—filler” composite, where the filled monolithic polymer is comprised of a spatial polymeric network of structural elements struts, nodes, walls and un-foamed volumes [27]. When producing a filled PU foam block, the clay filler is dispersed and exfoliated in polyol, and the dispersion is then mixed with the other components of the formulation (Table 1). The acquired liquid reacting mixture (LRM) of the polyurethane forming-substances (PFS) and the nanoclay filler is poured into a steel mould for foaming and the mould is sealed [3,25,26,27]. Let us estimate how the mass and volume of the monolithic polymer, and the gas in the cells and the filler change with an increase in the filler concentration.

The following main assumptions are made: (1) the mass of the LRM stays constant at any η and is equal to a pre-set value m_0_:

(7) $$m_{LRM}=m_{PFS}+m_{fil}=m_{0}=const.;$$

where m_PFS_—mass of the PFS and m_fil_—mass of the filler; (2) foaming takes place in a sealed mould (A delimited volume); and (3) the foams fill the inner volume of the mould V_0_ completely and uniformly. Shrinking of the produced blocks due to cooling and curing is not taken into account, meaning that the volume of the blocks stays constant and equal to the inner volume of the mould (if shrinking is substantial, it can be taken into account by an empiric relationship). Then, the apparent overall density (ISO 845:2006) of the filled PU foam ρ_foamfil_ in the blocks stays constant in any location of a block and at any concentration of the filler:

(8) $$\rho_{foamfil}=\frac{m_{0}}{V_{0}}=const.$$

### 3.2. Mass

When poured into the mould, the LRM foams and fills the inner volume V_0_ of the mould. In the result of chemical reaction, a monolithic polymer forms from the PFS and hardens between the gaseous bubbles. A polymeric network of structural elements (Struts, nodes, walls, and un-foamed volumes) is created in which the filler’s particles are incorporated. The mass losses of LRM due to chemical reactions and the release of gasses are neglected. The mass of the filler at a concentration η from the mass of the LRM is calculated:

(9) $$m_{fil}=\eta m_{LRM}=\eta m_{0}.$$

The mass of the filled PU foams in a moulded block equals the summary mass of its components: the neat (unfilled) monolithic polymer, the filler, and the gas in the cells:

(10) $$m_{foamfil}=m_{pol}+m_{fil}+m_{gas}$$

The mass of the gas in cells can be neglected as comparatively small:

(11) $$m_{gas}\ll m_{pol}\;\mathrm{and}\;m_{gas}\ll m_{fil},\;\mathrm{then}\;m_{gas}\approx 0.$$

Then the mass of filled PU foam in a block equals the mass of the LRM:

(12) $$m_{foamfil}=m_{LRM}=m_{PFS}+m_{fil}=m_{0}.$$

Formation of the monolithic polyurethane from the liquid PFS is assumed to happen without significant losses of mass. Then, the mass of the polymer remains the same as that of the PFS:

(13) $$m_{pol}=m_{PFS}=m_{0}-m_{fil}=m_{0}\left(1-\eta \right).$$

### 3.3. Volume

When the filler is fully exfoliated, the summary volume of the clay equals:

(14) $$V_{fil}=\frac{m_{fil}}{\rho_{fil}}=\eta \frac{m_{0}}{\rho_{fil}}$$

where ρ_fil_—density of the clay platelets. Density of the liquid PFS was determined experimentally as ρ_PFS_ = 1147 kg/m^3^ and density of the monolithic NEOpolyol-380 polyurethane was assumed ρ_pol_ ≈ 1280 kg/m^3^ [29]. It can be seen that ρ_pol_ > ρ_PFS_, meaning that at formation of the monolithic polymer, the volume occupied by PFS has shrunk. Using Equation (14), a linear relationship can be outlined between the volume of the liquid PFS and that of the monolithic polyurethane:

(15) $$V_{pol}=V_{PFS}\frac{\rho_{PFS}}{\rho_{pol}}=CV_{PFS};\;\mathrm{where}\;C=\frac{\rho_{PFS}}{\rho_{pol}}$$

The volume of the neat monolithic polyurethane equals:

(16) $$V_{pol}=\frac{m_{pol}}{\rho_{pol}}=\frac{m_{0}}{\rho_{pol}}$$

Then, the volume of the filled polymeric network is calculated as:

(17) $$V_{polfil}=V_{pol}+V_{fil}=\frac{m_{pol}}{m_{pol}}+\eta \frac{m_{0}}{\rho_{fil}}=m_{0}\left(\frac{1-\eta }{\rho_{pol}}+\frac{\eta }{\rho_{fil}}\right).$$

The difference between the volume of the neat polymeric network and that of the filled one is calculated as:

(18) $${∆V}_{pol,fil}=V_{pol}-V_{polfil}=\frac{m_{0}}{m_{pol}}-m_{0}\left(\frac{1-\eta }{\rho_{pol}}+\frac{\eta }{\rho_{fil}}\right)\;=\eta m_{0}\frac{∆\rho_{fil,pol}}{\rho_{fil}\rho_{pol}};\;\mathrm{where}\;∆\rho_{fil,pol}=\rho_{fil}-\rho_{pol}.$$

It can be seen when ρ_fil_ > ρ_pol_, then Δρ_fil,pol_ > 0 and ΔV_pol,fil_ > 0, a volume reduction of the polymeric network occurs when the density of the filler is higher than the density of the polymer. Including the densities of polymer and filler into a function F, the following relationship is derived:

(19) $${∆V}_{pol,fil}=\eta m_{0}F\left(\rho_{fil},\rho_{pol}\right);\;\mathrm{where}\;F\left(\rho_{fil},\rho_{pol}\right)=\frac{∆\rho_{fil,pol}}{\rho_{fil}\rho_{pol}}.$$

For a certain fixed polymer and filler, at a condition m_0_ = const., the volume reduction of the PU foam polymeric network due to filling is linearly proportional to the concentration of the filler. The relative reduction of volume R_ΔV_ of the volume of the neat monolithic polyurethane V_pol_ is expressed as:

(20) $$R_{∆V}=\frac{{∆V}_{pol,fil}}{V_{pol}}=\eta \frac{\rho_{fil}-\rho_{pol}}{\rho_{fil}}=\eta \frac{{∆\rho }_{fil,pol}}{\rho_{fil}}.$$

R_ΔV_ depends on density of filler, density of polymer and concentration of filler. It is independent of mass m_0_ and remains the same for filled PU foams of different densities, unless ρ_fil_, ρ_pol_ and η remain constant.

The volume of the gas in the cells equals the volume of the block minus the volume of the filled polymeric network:

(21) $$V_{g}=V_{0}-V_{polfil}=V_{0}-m_{0}\left(\frac{1-\eta }{\rho_{pol}}+\frac{\eta }{\rho_{fil}}\right).$$

The relative density γ and porosity ξ of the filled PU foams equals:

(22) $$\gamma =\frac{\rho_{foamfil}}{\rho_{polfil}}=\frac{V_{polfil}}{V_{0}}=\frac{m_{0}}{V_{0}\rho_{pol}}\left(1-\eta \frac{\rho_{fil}-\rho_{pol}}{\rho_{fil}}\right)\;\mathrm{and}$$

(23) ξ=1.0−γ.

### 3.4. Numerical Calculations of Components’ Characteristics

Numerical calculations of the characteristics of NEOpolyol-380 PU foam components (polymer, gaseous phase and filler), were performed at m_0_ = 250 g, for the actual dimensions of the inner volume of the mould: top 15 cm × 15 cm, bottom 14 cm × 14 cm, height 5 cm and volume V_0_ = 1052 cm^3^, density of the PFS ρ_PFS_ = 1147 kg/m^3^, density of the monolithic polyurethane ρ_pol_ ≈ 1280 kg/m^3^ and density of the nanoclay platelets ρ_fil_ = 3292 kg/m^3^ [19]. To probate the mathematical model, the mass and volume of the components as well as the relative density γ and porosity ξ of the filled PU foams were calculated in the full range of concentrations 0% ≤ η ≤ 100%. In practice, viscosity of the LRM increases rapidly already at η ≥ 5% and the highest impact on the physical/mechanical properties of PU foams is detected mainly at concentrations of nanoclay <5% [3,10,11,12,13,14,15,16,17]. Therefore, the characteristics of the PU foam components were calculated also for concentrations of filler η = 0%, 0.25%, 0.5%, 2%, 3% and 5% of the produced NEOpolyol-380 PU foam blocks.

It is known that the density ρ_pol_ of the neat monolithic polyurethane, which forms the polymeric network of PU foams, depends on the formulation, raw materials, production technologies, atmospheric conditions at polymerisation etc. [1,27]. The volume reduction of the polymeric network ΔV_pol,fil_ was calculated for different ratios ρ_fil_/ρ_pol_.

## 4. Results and Discussion

### 4.1. Results of Numerical Calculations

**Figure 2:**
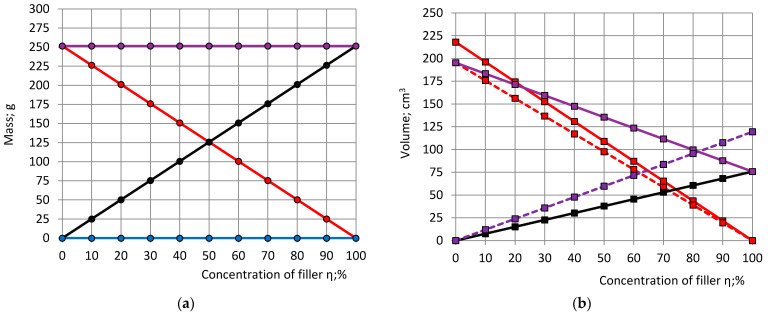
The dependence on concentration of filler η of: (**a**) mass of PFS/Polymer (Red), filler (Black), gas (Blue) and PU foam block/Filled polymer (Violet); (**b**) volume of PFS (Red), polymer (Red dashed), filler (Black), the filled polymer network (Violet) and volume reduction of the filled polymer network (Violet dashed).

The calculated characteristics, mass, and volume of the PU foam components are given in Figure 2. It can be seen, if ρ_fil_ > ρ_pol_ and the assumptions made in Point 3.1 are met, then at an increase in filler concentration from 0% to a 100%, the summary mass of the polymer and filler stays constant, but the summary volume decreases to around 39% of the initial V_polfil_. At filling, the volume of the polymeric network decreases for an amount ΔV_polfil_ and the volume of the gaseous phase increases for the same amount, which means that the porosity of the filled PU foams is higher than the porosity of the neat foams. The relative density decreases, and the porosity increases linearly with an increase in η. In limit cases, when (1) η = 0% (neat foams), γ = 18.6% and ξ = 81.4%; when (2) η = 100% (only filler), γ = 7.2% and ξ = 92.8%.

When the assumptions made in Point 3.1 are valid, the density of the nanoclay-filled PU foams in any location of any block equals ρ_0_ = m_0_/V_0_ ≈ 238 kg/m^3^.

Characteristics of PU foam components, calculated at practically useful concentrations of filler η = 0%, 0.25%, 0.5%, 2%, 3% and 5% as well as at η = 10%, are given in Table 2.

At an increase in filler concentration from 0% to 5% the relative density γ decreases from 18.6% to 17.4% and porosity ξ increases from 81.4% to 82.6%.

**Figure 3:**
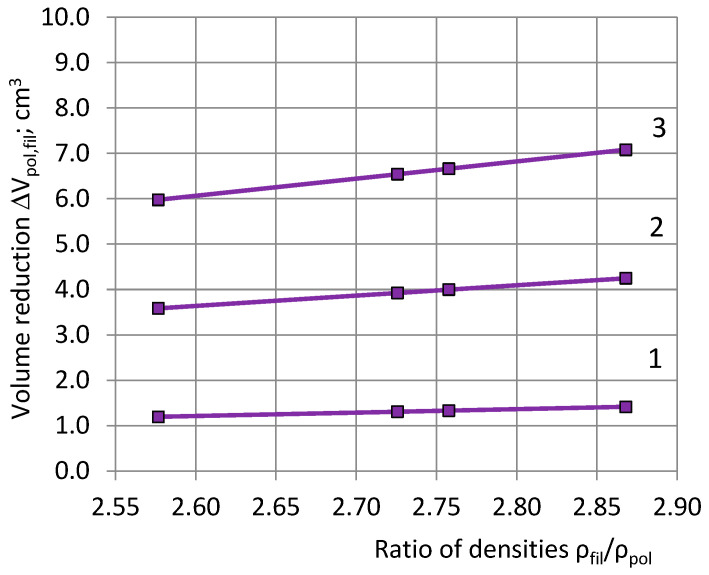
The volume reduction of polymeric network ΔV_pol,fil_ in PU foams dependent on the ratio of densities ρ_fil_/ρ_pol_ at filler concentrations of (1) 1%, (2) 3% and (3) 5%.

Table 3 and Figure 3 present the numerical results for the volume reduction of the polymeric network ΔV_pol,fil_ in PU foams dependent on the density ratio ρ_fil_/ρ_pol_, when PU foams of different formulations are filled with nanoclay platelets with a density of ρ_fil_ = 3292 kg/m^3^.

Volume reduction of the polymeric network can lead to reduced dimensions of the network elements (polymeric struts, nodes, walls, and un-foamed volumes). In addition, the nanoclay platelets act as nucleation sites of the gaseous bubbles, increasing the number of cells per unit volume and reducing size of structural elements [3,26,29], which can reduce PU foam stiffness and strength. The impact of the filling is determined by: (1) stiffening of the polymeric network due to being filled with nanoclay platelets; and (2) a reduction in the dimensions of the load-carrying elements. At practically efficient concentrations of η ≤ 5%, for: (1) petrochemical PU foams with a polyurethane density of ρ_pol_ ≈ 1280 kg/m^3^ volume reduction of polymeric network ΔV_pol,fil_ ≤ 5.97 cm^3^ and a relative volume reduction of R_ΔV_ ≤ 3.1% (Table 3); and for (2) rapeseed-oil polyol biofoams of ρ_pol_ ≈ 1150 kg/m^3^ ΔV_pol,fil_ ≤ 7.07 cm^3^ and R_ΔV_ ≤ 3.3% (Table 3). The resulting impact must be estimated for each PU foam, mass m_0_, filler, and concentration, individually.

### 4.2. Results of XRD Analysis

Characteristic changes were observed in the XR diffraction patterns [25,26]: (a) the angular position of the reflex 001 moved to smaller angles due to penetration of the macro chains into galleries; and (b) the intensity of the diffraction peak decreased because of the delamination of the nanoclay particles. It was concluded that the nanoclay Cloisite-30B had not fully exfoliated and that the intercalation dominated, as indicated by the still visible diffraction peaks.

### 4.3. Mass of NEOpolyol-380 PU Foam Blocks

In practice, deviations from the target mass m_0_ = 250 g appear in the technological process, which causes scattering of the actual mass of NEOpolyol-380 PU foam blocks at around m_0_ (Table 4).

**Figure 4:**
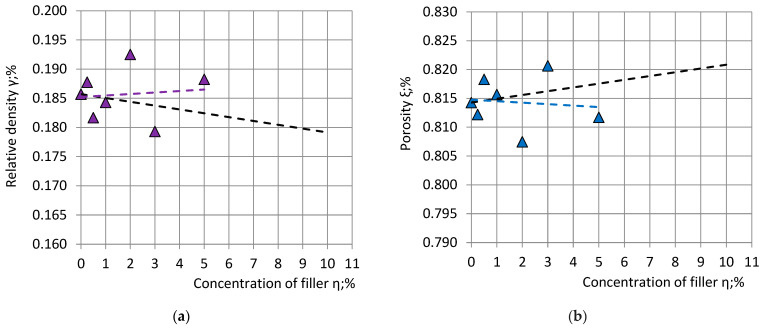
The dependence of: (**a**) relative density γ; and (**b**) porosity of filled NEOpolyol-380 PU foams on concentration of filler η. Markers—experimental data; the dashed straights: (1) violet and blue—trendlines of experimental data; and (2) black—theoretical estimations.

It can be seen that the relative difference between the target mass m_0_ = 250 g and the actual mass m of the seven produced NEOpolyol-380 PU foam blocks (244 g ≤ m ≤ 261 g) is ≤5%. The values of the blocks’ mass lie in a range of width of 261 g − 244 g = 17 g (≈20 g). Substituting the actual mass of the blocks into Equations (22) and (23), their relative density and porosity were calculated (Figure 4).

The scattering of the blocks’ mass is caused by: (1) variations in the mass of liquid reacting mixture poured into the mould, (2) uncertainties related to scale; (3) variations in the mass of the reacting mixture escaping through the gas-release holes; and (4) the subjective factor of the technologist, etc.

**Figure 5:**
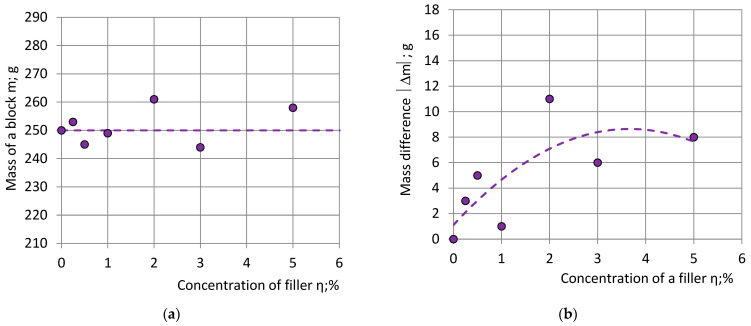
The dependence of: (**a**) mass of blocks m; and (**b**) the absolute value of mass difference |Δm| on concentration of filler η.

The dependence of block’s mass m and the absolute value of the mass difference |Δm| on the filler concentration η is given in Figure 5. The mass of the neat PU foam block, having the lowest viscosity of the liquid reacting mixture, is the closest to the target mass: m_1_ ≈ m_0_ = 250 g. Of the remaining six blocks, three (50%) had a mass higher than the target mass m > m_0_ and three (50%)—lower than m_0_: m < m_0_. With an increase in filler concentration in the liquid reacting mixture |Δm| increases as well, since the addition of the nanoclay filler increases the viscosity of the mixture [3,10,11,12,13,14,15].

### 4.4. Specimens from the Zone of Comparatively Uniform Density

The density of cubic specimens from Sections C-a and C-b of NEOpolyol-380 PU foam blocks is given in the Supplementary Materials, Table S2. The density range of the specimens from Section C-a is 219.5 kg/m^3^ ≤ ρ ≤ 230.4 kg/m^3^ and that of Section C-b is 216.7 kg/m^3^ ≤ ρ ≤ 229.1 kg/m^3^. The relative density difference of specimens from similar locations (1 and 1′, 2 and 2′, …, 5 and 5′) in Sections C-a and C-b is R ≤ 2%. The coefficient of variation of the density v ≤ 1% for all the blocks, for (a) 5 specimens from Section C-a, (b) 5 specimens from Section C-b and (c) 5 + 5 = 10 specimens from Sections C-a and C-b. It is concluded that the zone, enclosed by the green rectangles (Figure 1) is of a highly uniform density.

**Figure 6:**
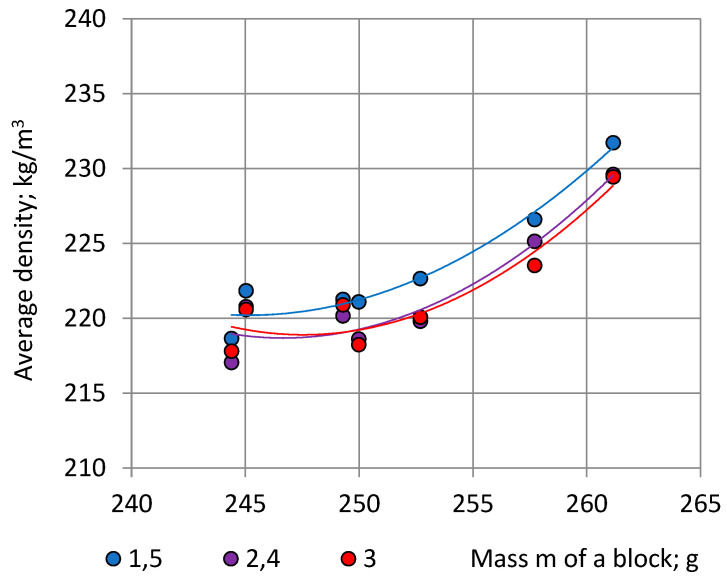
Average density of cubic specimens from symmetric locations in dependence of the mass of NEOpolyol-380 PU foam blocks. Section C-a specimens: ρ_1,5_—blue, ρ_2,4_—violet and density of the No 3 specimen ρ_3_—red. Trendlines: continuous lines.

In numerical calculations the second order polynomials (Function “Trendlines”, EXCEL) were applied for approximation of correlations “m—ρ”. First, the experimentally determined mass values (Table 4), lying in a range 244 g ≤ m ≤ 261 g, were used as input data, Figure 6. 

**Figure 7:**
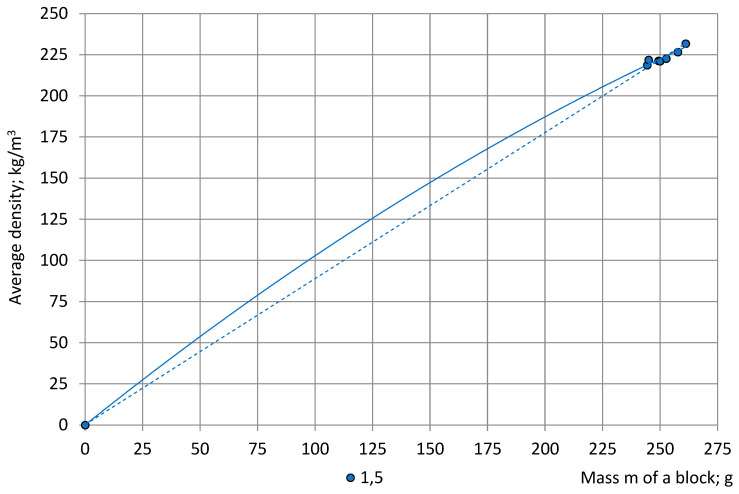
Average density of cubic specimens from symmetric locations in dependence of the mass of NEOpolyol-380 PU foam blocks; Section C-a specimens: ρ_1,5_—blue. Trendlines: continuous line—2-nd order polynomial and dashed line—linear function.

Analysis of the acquired trendlines

(24) $$\rho_{1,5}=0.0436m^{2}-21.3782m+2840.4012;\;R^{2}=0.9510;\;\;\;\rho_{2,4}=0.0517m^{2}-25.5197m+3366.1133;\;R^{2}=0.9137\;\mathrm{and}\;\rho_{3}=0.0537m^{2}-26.6058m+3511.9966;\;R^{2}=0.8940;$$

where R^2^—coefficient of correlation, shows that the trendlines (24) don’t match a basic condition: when m = 0 g, then ρ_av_ = 0 kg/m^3^. Therefore, the point m = 0 g; ρ = 0 kg/m^3^ was added to the experimental data. The correlation “m—ρ” was approximated with a 2-nd order polynomial and a linear function. Figure 7 gives, as an example, full-size graphs for the average density ρ_1,5_ of the Section C-a specimens (The graphs “ρ_2,4_—m” and “ρ_3_—m” are not given due to overlapping).

It can be seen that in the range 244 g ≤ m ≤ 261 g the linear function and the second order polynomial provide a similar slope to the axis of mass. The polynomial trendlines are considered further as more flexible in case of more data points. The trendline

(25) $$\rho_{1,5}=-0.0009m^{2}+1.1202m+0.0040;\;R^{2}=0.9996;$$

ensures matching the mentioned condition. The correlation “ρ_1,5_—m” is high: R^2^ = 0.9996. Similar results were acquired for correlations “m—ρ_2,4_” and “m—ρ_3_”. The following trendlines were determined for the Section C-a and Section C-b specimens:

(26) $$\rho_{1,5}=-0.0009m^{2}+1.1202m+0.0040;\;R^{2}=0.9996;\;\;\;\rho_{2,4}=-0.0010m^{2}+1.1427m+0.0049;\;R^{2}=0.9994\;\mathrm{and}\;\rho_{3}=-0.0013m^{2}+1.2121m+0.0049;\;R^{2}=0.9994;$$

(27) $$\rho_{1\prime ,5\prime }=-0.0009m^{2}+1.1203m+0.0047;\;R^{2}=0.9995;\;\;\;\rho_{2\prime ,4\prime }=-0.0009m^{2}+1.0972m+0.0056;\;R^{2}=0.9993\;\mathrm{and}\;\rho_{3\prime }=-0.0011m^{2}+1.1606m+0.0057;\;R^{2}=0.9994.$$

**Figure 8:**
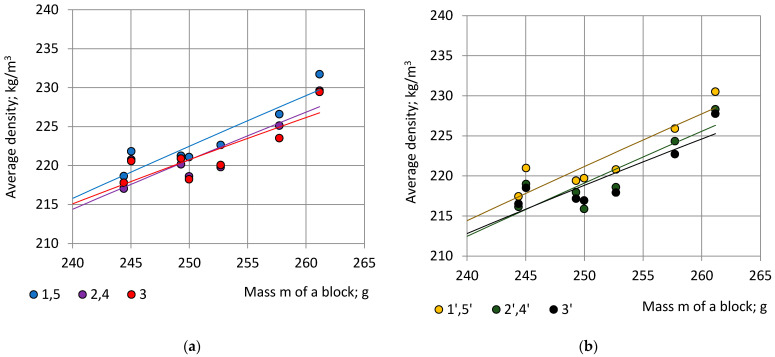
Average density of cubic specimens from symmetric locations in dependence of the mass of NEOpolyol-380 PU foam blocks: (**a**) section C-a specimens: ρ_1,5_—blue, ρ_2,4_—violet and density of the No 3 specimen ρ_3_—red; and (**b**) section C-b specimens: ρ_1′,5′_—yellow, ρ_2′,4′_—green and density of the No 3′ specimen ρ_3′_—black.

Correlation is high: 0.9993 ≤ R^2^ ≤ 0.9996. Since we are interested in the character of trendlines at mass values 244 g ≤ m ≤ 261 g, the corresponding part of the full-size graphs is given in Figure 8.

In the considered range of blocks’ mass 244 g ≤ m ≤ 261 g the relationship “m—ρ” is nearly linear. The average density of “Side” specimens 1 and 5 and 1′ and 5′ is the highest; density of the “Central” specimens 3 and 3′ is the lowest. It can be seen that the density of PU foams is not completely uniform even in the presumably uniform part of the block: the foams closer to the sides are denser than foams in the centre due to the non-adiabatic processes at the contact surfaces of the block with the mould [5,25].

Specimens in pairs 2, 4 and 1′, 5′ are in similar foaming conditions due to symmetric location to the plane X_2_OX_3_ in a block (Point 2.2). Specimens in pairs 2, 1′ and 4, 5′ are in similar foaming conditions due to symmetry to the diagonal axes O_1_O’_1_ and O_2_O’_2_ of a block, Figure 1a, consequently, the average densities ρ_2,4_ = ρ_1′,5′_, which is confirmed by the experimental data, Figure 8a,b. All locations at similar foaming conditions are given in Figure S1 of the Supplementary Materials.

The increase rate of the average density (Density in “Central” locations 3 and 3′) with respect to the blocks’ mass in the point m = m_0_ = 250 g is calculated in “Side” locations 1, 5 and 1′, 5′; “Intermediate” locations 2, 4 and 2′, 4′ as well as in “Central” locations 3 and 3′:

(28) $$\frac{d\rho_{1,5}}{dm}\approx 0.67\;\frac{kg}{m^{3}}/g,\;\frac{d\rho_{2,4}}{dm}\approx 0.64\;\frac{kg}{m^{3}}/g\;and\;\frac{d\rho_{3}}{dm}\approx 0.56\;\frac{kg}{m^{3}}/g;\;\frac{d\rho_{1\prime ,5\prime }}{dm}\approx 0.67\;\frac{kg}{m^{3}}/g,\;\frac{d\rho_{2\prime ,4\prime }}{dm}\approx 0.64\;\frac{kg}{m^{3}}/g\;and\;\frac{d\rho_{3}}{dm}\approx 0.61\;\frac{kg}{m^{3}}/g.$$

When the mass of a foam block differs from the target mass m_0_ = 250 g for Δm, the average densities in locations of Sections C-a and C-b differ from the average densities in the corresponding locations of a block with mass m = m_0_ = 250 g for Δρ = (dρ/dm)Δm. Let us estimate the density differences, when the difference of blocks’ mass Δm is a half of the blocks’ mass variation interval (20 g); Table 4. Then Δm = ± 10 g; R_n_ = Δm_n_/m_0_ = 4%, n = 1, 2, …, 7 and

Δρ_1,5_ = ± 6.7 kg/m^3^ (3.0%), Δρ_2,4_ = ± 6.4 kg/m^3^ (2.9%), Δρ_3_ = ± 5.6 kg/m^3^ (2.5%);
Δρ_1′,5′_ = ± 6.7 kg/m^3^ (3.0%), Δρ_2′,4′_ = ± 6.5 kg/m^3^ (3.0%), Δρ_3′_ = ± 6.1 kg/m^3^ (2.8%), (29)

where the relative density difference R_n_(ρ) = Δρ_n_/ρ(m_0_) = (m_n_ − m_0_)/m_0_.

**Figure 9:**
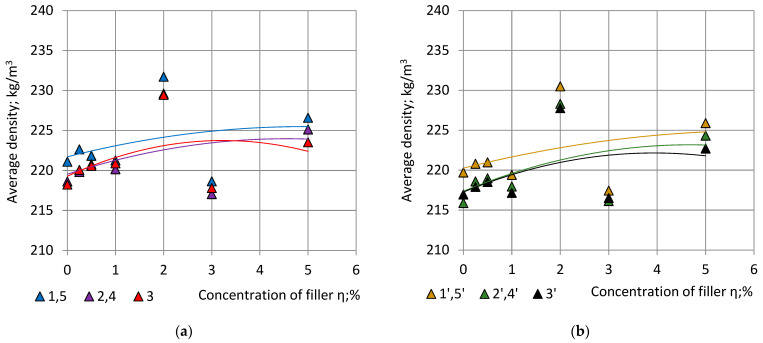
Average density of cubic specimens from symmetric locations in dependence of the filler’s concentration of NEOpolyol-380 PU foam blocks: (**a**) section C-a specimens: ρ_15_—blue, ρ_24_—violet and density of the № 3 specimen ρ_3_—red; and (**b**) section C-b specimens: ρ_1′5′_—yellow, ρ_2′4′_—green and density of the № 3′ specimen ρ_3′_—black.

The dependence of the average density of the specimens from similar locations of Section C-a and C-b on the concentration of the filler is given in Figure 9.

In the limits of considered concentrations η = 0% … 5% the relationship “η—ρ” was approximated with 2-nd order polynomials:

(30) $$\rho_{1,5}=-0.1558\eta^{2}+1.5350\eta +221.71;\;R^{2}=0.1116;\;\;\;\rho_{2,4}=-0.2141\eta^{2}+1.9493\eta +219.53;\;R^{2}=0.1606\;\mathrm{and}\;\rho_{3}=-0.4295\eta^{2}+2.7702\eta +219.28;\;R^{2}=0.1747;$$

(31) $$\rho_{1\prime ,5\prime }=-0.1235\eta^{2}+1.5270\eta +220.25;\;R^{2}=0.1424;\;\;\;\rho_{2\prime ,4\prime }=-0.2663\eta^{2}+2.4910\eta +217.35;\;R^{2}=0.2440\;\mathrm{and}\;\rho_{3\prime }=-0.3163\eta^{2}+2.4903\eta +217.25;\;R^{2}=0.2124.$$

Correlation “η—ρ” between density of the cubic specimens and the concentration of the filler of PU foam blocks is low: 0.11% ≤ R^2^ ≤ 25%. Due to the C_4_ rotational symmetry around axis OX_3_ of the structure of PU foam blocks, the outlined relationships “m—ρ” and “η—ρ” remain valid for locations, which correspond to rotations of Sections C-a and C-b for angles 90°, 180° and 270° around the axis OX_3_, Figure 1.

Since the dependence of the foam density on the concentration is insignificant, it can be concluded, if the mass of all the 7 blocks (η = 0%, 0.25%, 0.5%, 1%, 2%, 3% and 5%); would be ideally equal to the target mass m_0_ = 250 g, PU foam density in similar locations of the blocks and produced in a sealed mould would be equal too. The eventual differences would be determined by other factors like local fluctuations in chemical reactions, thermal conditions etc. Practically various technological factors (Point 4.3) hinder the equality of the blocks’ mass even at equal concentrations of filler.

### 4.5. Specimens from the Side of Blocks

Density distribution in the Section S is given in Supplementary Materials, Tables S3–S12. The average density of the 16 perimetral specimens exceeds that of the 9 central specimens for 9 kg/m^3^–18 kg/m^3^ (4–8%). The average density of the nine central (16 perimetral) specimens exceeds that of the adjacent cubic specimens 2, 3 and 4 from Section C-a for 10 kg/m^3^–14 kg/m^3^ (4–6%). The average density at the five bottom specimens of Section S is up to 20 kg/m^3^ higher than of the five top specimens.

**Figure 10:**
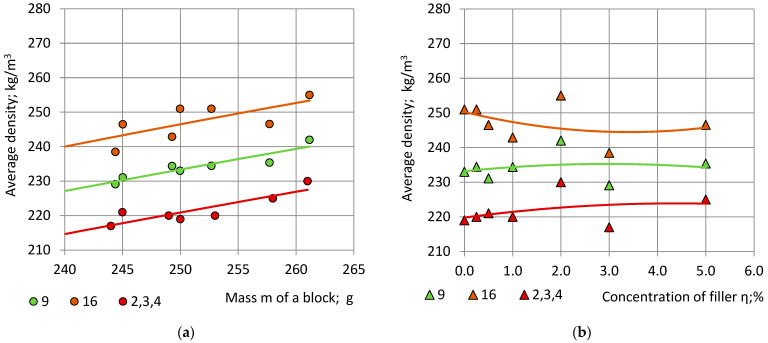
Average density of the nine central side specimens ρ_SC9_—green, and of the 16 perimetral side specimens ρ_SP16_—orange, from Section S and of the cubic specimens No 2, 3 and 4 from Section C-a—bordo in dependence of: (**a**) the mass of NEOpolyol-380 PU foam blocks; and (**b**) the filler’s concentration of NEOpolyol-380 PU foam blocks.

Dependence of the average density of the 16 perimetral and of the 9 central specimens of the Section S on the mass of PU foam blocks is given in Figure 10 together with the average density in the adjacent cubic specimens 2, 3 and 4 from Section C-a.

The point m = 0 g and ρ = 0.0 kg/m^3^ was added to the experimental data to ensure passing of the graph “m—ρ” through the “0” point. The relationship “m—ρ” for the average density of the 16 perimetral and of the 9 central specimens was approximated with second order polynomials:

(32) $$\rho_{16}=-0.0014m^{2}+1.3384m-0.0019;\;R^{2}=0.9983;\;\;\rho_{9}=-0.0013m^{2}+1.2545m+0.0013;\;R^{2}=0.9997\;\mathrm{and}\;\rho_{2-4}=-0.0011m^{2}+1.1532m+0.0049;\;R^{2}=0.9993.$$

In the considered range of mass 244 g ≤ m ≤ 261 g the relationship “m—ρ” is nearly linear, Figure 8a. The increase rate of the average density with respect to the blocks’ mass is similar in “Side” locations 1, 5 and 1′, 5′; “Intermediate” locations 2, 4 and 2′, 4′ and “Central” locations 3, 3′:

(33) $$\frac{d\rho_{16}}{dm}\approx 0.63\frac{kg}{m^{3}}/g,\;\frac{d\rho_{9}}{dm}\approx 0.60\frac{kg}{m^{3}}/g\;and\;\frac{d\rho_{2-4}}{dm}0.60\approx \frac{kg}{m^{3}}/g.$$

Density increase rate is the highest for the 16 perimetral side specimens. The value of dρ_2–4_/dm of the cubic specimens 2, 3 and 4 from the Section C-a is similar to the values of dρ_2,4_/dm and dρ_3_/dm in the Section C-a.

Within the limits of the considered concentrations η = 0% … 5%, the correlation “η—ρ” was approximated with second order polynomials:

(34) $$\rho_{16}=0.4987\eta^{2}-3.4006\eta +250.26;\;R^{2}=0.1552;\;\;\rho_{9}=-0.2377\eta^{2}+1.4044\eta +233.18;\;R^{2}=0.0351\;\mathrm{and}\;\rho_{2-4}=-0.2143\eta^{2}+1.8760\eta +219.77;\;R^{2}=0.1343.$$

It can be seen that, at concentrations 0% … 5% the correlation “η—ρ” between the density of the side specimens and the filler concentration of the PU foam blocks is low: 3% ≤ R^2^ ≤ 16%. The relationships “m—ρ” and “η—ρ” remain valid for locations which correspond to rotations of Section S for 90°, 180° and 270° around the axis OX_3_ (Figure 1).

### 4.6. Experimental Data and Theoretical Estimations

Taking into account the experimentally detected insignificant correlation of specimens’ density with filler concentrations of 0.0% ≤ η ≤ 5.0%, the following conclusions can be made: (1) in a batch of foam blocks, the density differences of the specimens from similar locations are caused by scattering in the blocks’ mass, which increases with an increase in the viscosity of the liquid reacting mixture; and (2) the density differences in the PU foam specimens from different locations of a single foam block are caused by a non-uniform distribution of mass due to the heat exchange at the contact surfaces with the metallic mould.

**Figure 11:**
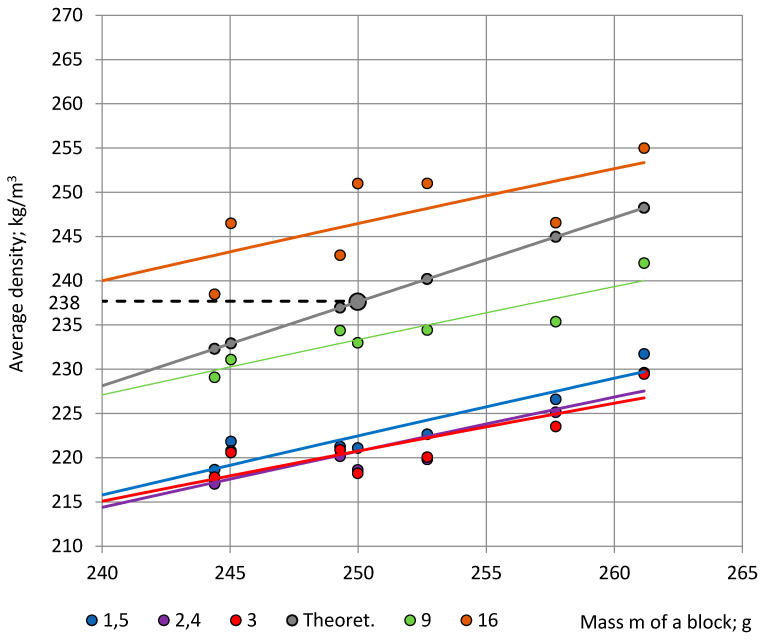
Average density of NEOpolyol-380 PU foams in dependence of the mass of foam blocks: (1) specimens from symmetric locations in the Section C-a: ρ_1,5_—blue, ρ_2,4_—violet and ρ_3_–red; (2) specimens from the Section S: 9 central specimens ρ_SC9_—green and 16 perimetral specimens ρ_SP16_—orange. Theoretical estimations of density in any location at: (1) a uniform distribution of mass—grey; and (2) a uniform distribution of mass, when mass of all blocks equals m_0_ = 250 g—the grey, bigger marker.

In this connection, two idealised cases can be considered at any concentration 0.0% ≤ η ≤ 5.0%: (1) the mass of all blocks is equal to the target mass m_0_ (no technological flaws); then, the density in different locations of a block equals that of the block with the mass m = m_0_; and (2) the mass of the liquid reacting mixture distributes uniformly in each block (no heat exchange); then, the density in any location of a block equals ρ_n_ = m_n_/V_0_; n = 1, 2, …, 7. The first case corresponds to the first theoretical assumption made in Point 3.1 and the second—to the third one. When both assumptions take place simultaneously, the density in any location of any block equals ρ_0_ = m_0_/V_0_ ≈ 238 kg/m^3^ (Figure 11, the grey, bigger marker).

In the production of blocks, a part of the liquid reacting mixture contacts the metallic mould and a heat exchange occurs which slows foaming. The heat exchange rate depends on the temperature difference between the reacting mixture and the external environment [22,23]. A skin and a layer of comparatively high-density foams forms. The corresponding mass does not participate in the mass distribution by overpressure in the major volume of a block [31,32,33]; therefore, the increase rate of the density in the major volume of the blocks is lower than what is theoretically possible.

Figure 11 depicts experimental data of the average density of specimens from Section C-a and Section S along with the theoretical estimates (data from Section C-b are not shown due to overlapping). The theoretical density value of 238 kg/m^3^ provides a rough estimate of the average density in the blocks. It can be seen that the experimental data are situated around the trendline of the theoretical data.

## 5. Conclusions

Mass and volume of nanoclay-filled medium-density NEOpolyol-380 PU foam components—polymer, gaseous phase, and filler, were estimated in a full range of filler concentrations 0% < η < 100%, when the foam was made in a sealed mould.

The mathematical model revealed a volume reduction of the nanoclay-filled polyurethane network, where the density of the nanoclay platelets was higher than the density of the polymer, which is commonly the case for polyurethanes. At the practically efficient concentrations of nanoclay Cloisite-30B η ≤ 5%, for petrochemical PU foams with a density of polyurethane of 1280 kg/m^3^ and for rapeseed-oil polyol biofoams with a density of polyurethane of 1150 kg/m^3^, the volume reduction was ≤5.97 cm^3^ (7.07 cm^3^) and the relative volume reduction was ≤3.1% (3.3%). The model, in principle, remains valid for elastic PU foams, for the relative densities of PU foams 0% … 100% as well as for the actual values of the mass of PU foam blocks.

Numerical calculations showed that the correlation “m—ρ” between the density of the specimens and mass of the blocks is high: in the uniform zone as well as in the side section the coefficient of correlation was 93% ≤ R^2^ ≤ 97%. The point (m = 0 g; ρ = 0 kg/m^3^) must be added to the experimental data to ensure passing of the trendlines through the point “0”. Correlation “m—η” between the density of specimens and the filler concentration insignificant with regard to the uniform zone 11% ≤ R^2^ ≤ 25% and the side section 3% ≤ R^2^ ≤ 16%.

Future research is necessary for the mathematical modelling and numerical simulation of nanoclay-filled PU foam mass distribution in a sealed mould, which takes into account the filler concentration, overpressure, and temperature of the external environment etc.

## Figures and Tables

**Table 1:** PU foam formulation.

Formulation of Polyol; Pbw *		
1	Recycled APP NEOpolyol-380	80.0
2	Cross-linking agent, Lupranol 3422	20.0
3	Flame retardant, TCPP	20.0
4	Blowing agent, water	1.0
5	Reactive catalyst, PC CAT NP 10	1.6
6	Surfactant, NIAX Silicone L6915	2.0
Polyisocyanate; pbw		193
Characteristics of formulation		
1	Recycled materials in PU foams,%	15
2	Isocyanate index	160
Technological parameters		
1	Cream time, s	25
2	String time, s	45
3	Tack-free time, s	60
4	Foaming end time, s	60

* Parts by weight per hundred parts of polyol.

**Table 2:** Characteristics of the components of PU foam at concentration of filler η = 10% (ΔV_pol,fil_—volume reduction of polymeric network).

η; %	Mass of Components; g			Volume of Component; cm^3^					ΔV_pol,fil_/V_polfil_; %	Relat. Density γ; %	Porosity ξ; %
	PFS; m_PFS_	Polymer; m_pol_	Filler; m_fil_	PFS; V_PFS_	Polymer; V_pol_	Filler; V_fil_	Filled Polymer; V_polfil_	ΔV_pol,fil_			
0.00	250.0	250.0	0.00	218.02	195.31	0.00	195.31	0.00	0.0	18.6	81.4
0.25	249.4	249.4	0.63	217.48	194.82	0.19	195.01	0.30	0.2	18.5	81.5
0.50	248.8	248.8	1.25	216.93	194.34	0.38	194.72	0.60	0.3	18.5	81.5
1.00	247.5	247.5	2.50	215.84	193.36	0.76	194.12	1.19	0.6	18.5	81.5
2.00	245.0	245.0	5.00	213.66	191.41	1.52	192.93	2.39	1.2	18.3	81.7
3.00	242.5	242.5	7.50	211.48	189.45	2.28	191.73	3.58	1.8	18.2	81.8
5.00	237.5	237.5	12.50	207.12	185.55	3.80	189.34	5.97	3.1	18.0	82.0
10.00	225.0	225.0	25.00	196.22	175.78	7.50	183.38	11.94	6.1	17.4	82.6

**Table 3:** The volume reduction of the polymeric network ΔV_pol,fil_ in PU foams dependent on the density ratio ρ_fil_/ρ_pol_.

PU Foams	Density of Monolit. PU ρ_pol_; kg/m^3^	Ratio of Densities ρ_fil_/ρ_pol_	Concentration of Filler η; %							
			0.25	0.50	1.00	2.00	3.00	4.00	5.00	10.00
			Volume Reduction of Polymeric Network ΔV_pol,fil_; cm^3^							
Petrochemical [21,27]	1 280	2.58	0.30	0.60	1.19	2.39	3.58	4.77	5.97	11.94
Petrochemical [3]	1 210	2.73	0.33	0.65	1.31	2.61	3.92	5.23	6.53	13.07
Petrochemical [30]	1 196	2.76	0.33	0.67	1.33	2.66	3.99	5.32	6.65	13.31
Biofoams [27]	1 150	2.87	0.35	0.71	1.41	2.83	4.24	5.66	7.07	14.14

**Table 4:** Characteristics of the produced NEOpolyol-380 PU foam blocks.

Number of a Block n	Concentration of Filler η; %	Mass m; g	Difference Δm; g	Relative Difference R_n0_; %	Relative Density γ; %	Porosity ξ; %
1	0.00	250	0	0.0	0.186	0.814
2	0.25	253	3	1.2	0.188	0.812
3	0.50	245	−5	−2.0	0.182	0.818
4	1.00	249	−1	−0.4	0.184	0.816
5	2.00	261	11	4.4	0.193	0.807
6	3.00	244	−6	−2.4	0.179	0.821
7	5.00	258	8	3.2	0.188	0.812

## Data Availability

Data are contained within the article.

## Supplementary Materials

The following supporting information can be downloaded at: https://www.mdpi.com/article/10.3390/polym15153228/s1, Table S1. Specimens in the Section S: (a) The 9 central specimens are located at the intersection of layers 2, 3, 4 and columns 2, 3, 4 (Enclosed by the ellipse) and (b) The 16 perimetral specimens are located in columns 1, 5 and layers 1, 5. Table S2. Density of cubic specimens from Sections C-a and C-b (In order of an increase of blocks’ mass). “AVERAGE”—average value, “σ”—standard deviationσ and “v”—coefficient of variation. Table S3. Denotations of density of PU foams’ specimens from the Section S. Tables S4–S10. Density distribution in the Section S of the blocks (In order of an increase of blocks’ mass). Table S11. Average density of the side specimens in Section “S”and cubic specimens in Section C-a (In order of an increase of the blocks’ mass). “σ”—standard deviation and “v”—coefficient of variation. Table S12. Density difference and relative density difference of the side specimens in Section “S”and cubic specimens in Section C-a (In order of an increase of the blocks’ mass). Figure S1. Locations A, B, C, D, E and F of specimens at similar foaming conditions in a PU foams’ block: A—1 and 5 (4 specimens); B—2, 4 and 1′, 5′ (8 specimens); C—3 (4 specimens); D—2′ (4 specimens), 4′; E—3′ (4 specimens) and F—the central specimen.
