# Supplementary material for: Characteristics of Components and Density of Rigid Nanoclay-Filled Medium-Density Polyurethane Foams Produced in a Sealed Mould
Source: Polymers (Basel). 2023 Jul 28;15(15):3228. doi: 10.3390/polym15153228 (PMC10422265; doi:10.3390/polym15153228)
Supplement: Supplementary file 1 [file polymers-15-03228-s001.zip › polymers-2505480-supplementary.pdf]

Characteristics of Components and Density of Rigid Nanoclay-Filled Medium-Density Polyurethane Foams Produced in a Sealed Mold

Ilze Beverte, Ugis Cabulis, Janis Andersons, Mikelis Kirpluks, Vilis Skruls and Peteris Cabulis

Supplementary Materials

Table S1. Specimens in the Section S: a) The 9 central specimens are located at the intersection of layers 2, 3, 4 and columns 2, 3, 4 (Enclosed by the ellipse) and b) The 16 perimetral specimens are located in columns 1, 5 and layers 1, 5.

Layers	Columns				
	5	4	3	2	1
5	5-5	5-4	5-3	5-2	5-1
4	4-5	4-4	4-3	4-2	4-1
3	3-5	3-4	3-3	3-2	3-1
2	2-5	2-4	2-3	2-2	2-1
1	1-5	1-4	1-3	1-2	1-1

Table S2. Density of cubic specimens from Sections C-a and C-b (In order of an increase of blocks' mass). "AVERAGE" – average value, "σ" - standard deviationσ and "ν" – coefficient of variation.

Mass of a block m_n; g	Concentr. of filler; %	Number of a block	Section C-a		Section C-b		Difference of density $\Delta\rho_{ij}$; kg/m³	Relative difference of density R_{ij}; %	Average density ρ_{av}; kg/m³
			Number of a specimen	ρ; kg/m³	Number of a specimen	ρ; kg/m³			
244	3.00	6	1	218.5	1′	218.0	0.5	0	218.3
			2	216.4	2′	216.1	0.3	0	216.3
			3	217.8	3′	216.5	1.3	1	217.2
			4	217.7	4′	216.2	1.5	1	216.9
			5	218.8	5′	216.9	1.9	1	217.8
245	0.50	3	1	221.5	1′	221.9	- 0.4	0	221.7
			2	221.0	2′	219.1	1.8	1	220.1
			3	220.6	3′	218.5	2.1	1	219.6
			4	220.6	4′	218.8	1.7	1	219.7
			5	222.2	5′	220.1	2.1	1	221.1
249	1.00	4	1	221.2	1′	220.2	1.0	0	220.7
			2	219.4	2′	218.6	0.8	0	219.0
			3	220.9	3′	217.2	3.7	2	219.0
			4	220.9	4′	217.3	3.6	2	219.1
			5	221.3	5′	218.6	2.7	1	220.0
250	0.00	1	1	220.9	1′	220.3	0.6	0	220.6
			2	219.3	2′	216.3	2.9	1	217.8
			3	218.3	3′	216.9	1.3	1	217.6

			4	218.0	4′	215.5	2.5	1	216.7
			5	221.3	5′	219.2	2.1	1	220.3
253	0.25	2	1	223.3	1′	221.7	1.6	1	222.5
			2	220.0	2′	219.0	0.9	0	219.5
			3	220.1	3′	217.9	2.2	1	219.0
			4	219.7	4′	218.2	1.5	1	218.9
			5	222.0	5′	219.9	2.1	1	221.0
258	5.00	7	1	226.5	1′	226.3	0.3	0	226.4
			2	224.9	2′	224.4	0.5	0	224.7
			3	223.5	3′	222.7	0.8	0	223.1
			4	225.3	4′	224.2	1.1	1	224.8
			5	226.7	5′	225.5	1.1	1	226.1
261	2.00	5	1	230.8	1′	231.4	- 0.5	0	231.1
			2	230.0	2′	228.7	1.3	1	229.3
			3	229.5	3′	227.8	1.7	1	228.6
			4	229.3	4′	228.0	1.3	1	228.6
			5	232.6	5′	229.7	3.0	1	231.1
			AVERAGE =	222.3	AVERAGE =	220.8			
			σ =	± 4.1	σ =	± 4.4			
			v =	1.9 %	v =	2.0 %			
	Sections C-a + C-b		AVERAGE =	221.5					
			σ =	± 4.3					
			v =	1.9 %					

Difference of densities of the cubic specimens from similar locations (1 and 1′, 2 and 2′, 3 and 3′, 4 and 4′ and 5 and 5) in the Sections C-a and C-b:

$\Delta\rho_{ij} = \rho_i - \rho_j$, (1)

where ρ_i and ρ_j – density of the i-th and j-th cubic specimens from similar locations in the Sections C-a and C-b; i = 1, 2, 3, 4 and 5 and j = 1′, 2′, 3′, 4′ and 5′.

The relative density difference of the i-th and j-th cubic specimens from similar locations in the Sections C-a and C-b; i = 1, 2, 3, 4 and 5 and j = 1′, 2′, 3′, 4′ and 5′:

$R_{ij} = \Delta\rho_{ij}/\rho_i = (\rho_i - \rho_j)/\rho_i$. (2)

Table S3. Denotations of density of PU foams′ specimens from the Section S.

Number of range of density	Range of density; kg/m^3	Shade
1	$210 \le \rho \le 220$ kg/m^3	
2	$220 < \rho \le 230$ kg/m^3	
3	$230 < \rho \le 240$ kg/m^3	
4	$240 < \rho \le 250$ kg/m^3	
3	$250 < \rho \le 260$ kg/m^3	
6	$260 < \rho \le 270$ kg/m^3	
7	$270 < \rho \le 280$ kg/m^3	

Tables S4 - S10. Density distribution in the Section S of the blocks (In order of an increase of blocks' mass).

Table S4. Mass of the block m = 244 g; block № 6.

	5	4	3	2	1
5	239	240	232	240	239
4	234	236	224	236	234
3	236	229	224	229	236
2	235	228	229	228	235
1	249	241	236	241	249

Table S5. Mass of the block m = 245 g; block № 3.

	5	4	3	2	1
5	248	241	241	241	248
4	241	230	233	230	241
3	242	227	228	227	242
2	244	235	232	235	244
1	258	251	252	251	258

Table S6. Mass of the block m = 249 g; block № 4.

	5	4	3	2	1
5	243	238	234	238	243
4	238	234	242	234	238
3	241	234	235	234	241
2	240	233	231	233	240
1	258	246	245	246	258

Table S7. Mass of the block m = 250 g; block № 1.

	5	4	3	2	1
5	256	254	255	254	256
4	242	237	235	237	242
3	240	231	230	231	240
2	244	234	231	234	244
1	258	258	257	258	258

Table S8. Mass of the block m = 253 g; block № 2.

	5	4	3	2	1
5	255	257	256	257	255
4	242	238	237	238	242
3	241	232	231	232	241
2	243	235	231	235	243
1	259	257	255	257	259

Table S9. Mass of the block m = 258 g; block № 7.

	5	4	3	2	1
5	242	239	233	239	242
4	243	232	232	232	243
3	241	243	234	243	241
2	245	235	232	235	245
1	264	254	256	254	264

Table S10. Mass of the block m = 261 g; block № 5.

	5	4	3	2	1
5	255	255	257	255	255
4	242	239	237	239	242
3	242	243	245	243	242
2	249	243	243	243	249
1	275	261	266	261	275

Table S11. Average density of the side specimens in Section "S"and cubic specimens in Section C-a (In order of an increase of the blocks' mass). "σ" – standard deviation and "v" – coefficient of variation.

Mass of a block m; g	Concentration of filler η; %	Average density ± σ; kg/m³ (v; %)		
		Section S		Section C-a
		ρ_{16}	ρ_9	ρ_{2-4}
244	3.00	238 ± 5 (2)	229 ± 4 (2)	217 ± 1 (0)
245	0.50	247 ± 6 (2)	231 ± 3 (1)	221 ± 0 (0)
249	1.00	243 ± 7 (3)	234 ± 3 (1)	220 ± 1 (0)
250	0.00	251 ± 7 (3)	233 ± 3 (1)	219 ± 1 (0)
253	0.25	251 ± 8 (3)	234 ± 3 (1)	220 ± 0 (0)
258	5.00	247 ± 9 (4)	235 ± 5 (2)	225 ± 1 (0)
261	2.00	255 ± 11 (4)	242 ± 3 (1)	230 ± 0 (0)

Table S12. Density difference and relative density difference of the side specimens in Section "S"and cubic specimens in Section C-a (In order of an increase of the blocks' mass).

Mass of a block m; g	Concentration of filler η; %	Density difference; kg/m³			Relative density difference; %		
		$\Delta\rho_{16,9}$	$\Delta\rho_{9,2-4}$	$\Delta\rho_{16,2-4}$	$R_{16,9}$	$R_{9,2-4}$	$R_{16,2-4}$
244	3.00	9	12	21	4	6	10
245	0.50	16	10	26	7	5	12
249	1.00	9	14	23	4	6	10
250	0.00	18	14	32	8	6	15
253	0.25	17	14	31	7	6	14
258	5.00	12	10	22	5	4	10
261	2.00	13	12	25	5	5	11

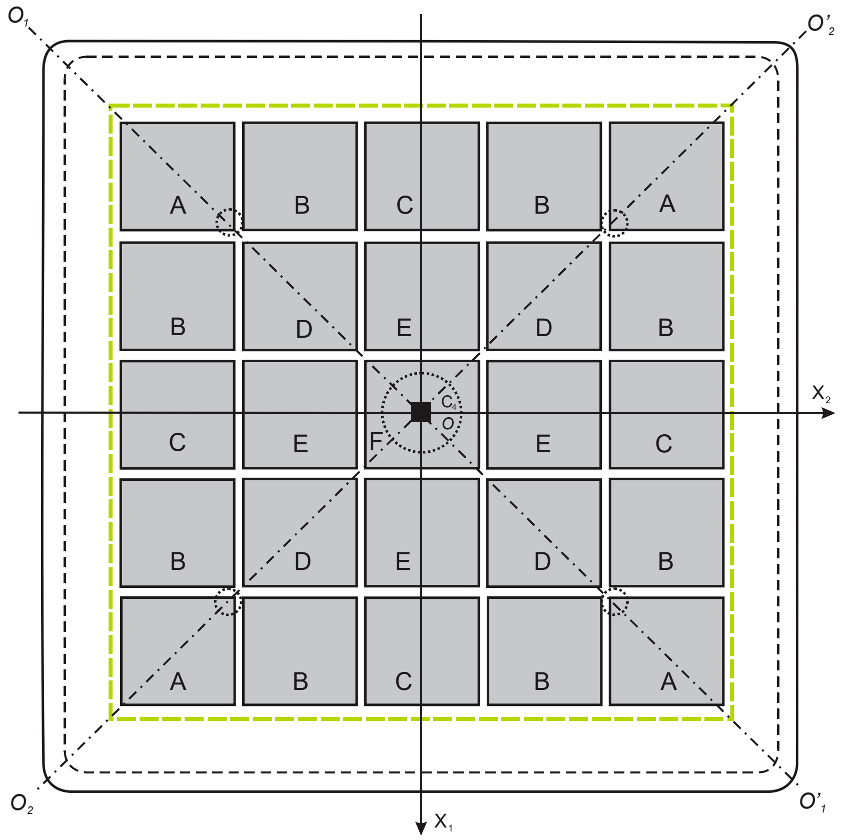

Figure S1. Locations A, B, C, D, E and F of specimens at similar foaming conditions in a PU foams' block: A - 1 and 5 (4 specimens); B - 2, 4 and 1', 5' (8 specimens); C – 3 (4 specimens); D - 2' (4 specimens), 4'; E - 3' (4 specimens) and F - the central specimen.
